# Half-Electrolysis of Water with the Aid of a Supercapacitor Electrode

**DOI:** 10.1021/acsaem.3c00615

**Published:** 2023-05-19

**Authors:** Yao Chen, George Zheng Chen

**Affiliations:** † The State Key Laboratory of Refractories and Metallurgy, College of Materials and Metallurgy, Wuhan University of Science and Technology, Wuhan 430081, P. R. China; ‡ Department of Chemical and Environmental Engineering, Faculty of Engineering, University of Nottingham, Nottingham NG2 7RD, U.K.

**Keywords:** half-electrolysis, electrolysis of water, stepwise, supercapacitor electrode, diaphragm

## Abstract

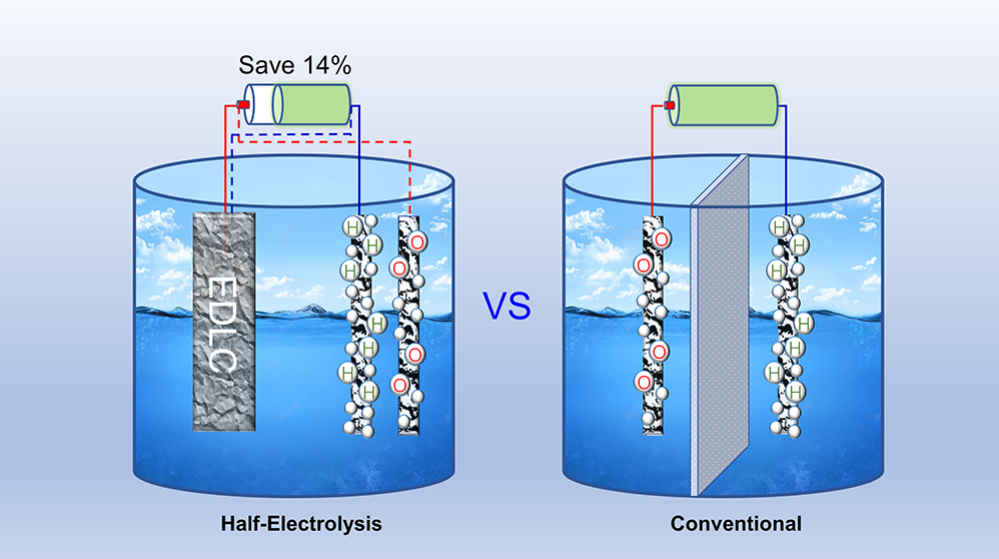

Half-electrolysis runs one desirable half-cell reaction with the aid of a counter supercapacitor electrode which replaces the other unwanted half-cell reaction occurred inevitably in conventional electrolysis. Herein, it is developed to complete the whole cell reaction of water electrolysis, in alternative steps, with a capacitive activated carbon (AC) electrode and an electrolysis Pt electrode. When positively charging the AC electrode, a hydrogen evolution reaction occurs at the Pt electrode. By reversing the current, the charge stored in the AC electrode is discharged to assist the oxygen evolution reaction on the same Pt electrode. Consecutive completion of the two processes realizes the overall reaction of water electrolysis. This strategy leads to stepwise production of H_2_ and O_2_ without the need of a diaphragm in the cell and hence results in a lower energy consumption compared with the practical conventional electrolysis.

## Introduction

The overall cell reaction of water electrolysis is divided into two half-cell reactions, namely, the hydrogen evolution reaction (HER) on the cathode and the oxygen evolution reaction (OER) on the anode. Hydrogen can be fed into fuel cells to generate electricity,^1−3^ while high-purity oxygen remains a crucial therapy of Covid-19-infected patients.^4^ All currently available electrolyzers require a minimum of potable-grade water. However, the scarcity of fresh water in some coastal arid zones of the world and marine industry make it more desirable to electrolyze seawater.^5−7^

Practically, a diaphragm is inevitably applied to separate H_2_ and O_2_ gases produced in the cathode and anode,^8,9^ which engenders at least five disadvantages. First, the diaphragm resistance brings about extra voltage loss and energy consumption increase with the applied current. Second, peculiar polymer membranes, such as the proton-exchange membrane (PEM)^10^ and the polysulfone membrane,^11^ are extraordinarily expensive. Third, gases can permeate through the diaphragm if the pressures on the anode and cathode sides of the cell are not well balanced because the volume of the produced H_2_ is in theory twice that of O_2_.^12^ Fourth, pressure balance restricts the applied electrolysis current and rate. Last but not least, a low pH electrolyte favors HER, but a high pH one is preferred for OER, while the currently available diaphragms are either acidic or alkaline only in nature.

In 2013, decoupling HER and OER during the electrolysis of water was realized with a phosphomolybdic acid.^13^ Since 2016, separations of HER and OER had been developed in the electro- and photoelectron water splitting without the membranes by faradaic reaction solid electrodes.^14−21^ In 2014, half-electrolysis was demonstrated to run a desirable half-cell reaction on an electrolysis electrode with the aid of a counter supercapacitor electrode,^22^ which only experienced charging and discharging, eliminating the other unwanted but inevitable half-cell reaction in conventional electrolysis. Typically, OER, even possible chlorine evolution reaction (ClER), for seawater electrolysis is not as important as HER for the hydrogen economy. As a result, the energy consumption for the desired product can be remarkably reduced in half-electrolysis. The challenge to the half-electrolysis is how to continue the process after the supercapacitor electrode is fully charged.

Herein, we demonstrated continuous water splitting via half-electrolysis with separated HER and OER in a cell that typically had a supercapacitor electrode of activated carbon (AC), an electrolysis electrode of Pt wire, and an electrolyte of simulated seawater. Different from separation of HER and OER with solid electrodes by faradaic reactions,^14^ it should be stressed that only one faradaic reaction occurred during each step to realize the real “half-electrolysis” because the AC electrode, different from those materials with faradaic CV peaks,^23−27^ stores the electric energy by a physical electric double-layer mechanism, which is expected to increase the applied current and prolong the lifetime of the instrument. When the AC electrode was charged, HER occurred on the Pt electrode as a cathode, while AC discharged by reversing the current, OER on the same Pt electrode as an anode. Because HER and OER did not occur simultaneously, the diaphragm was omitted in the half-electrolysis cell, overcoming the drawbacks of a diaphragm-based cell.

## Results and Discussion

**Figure 1:**
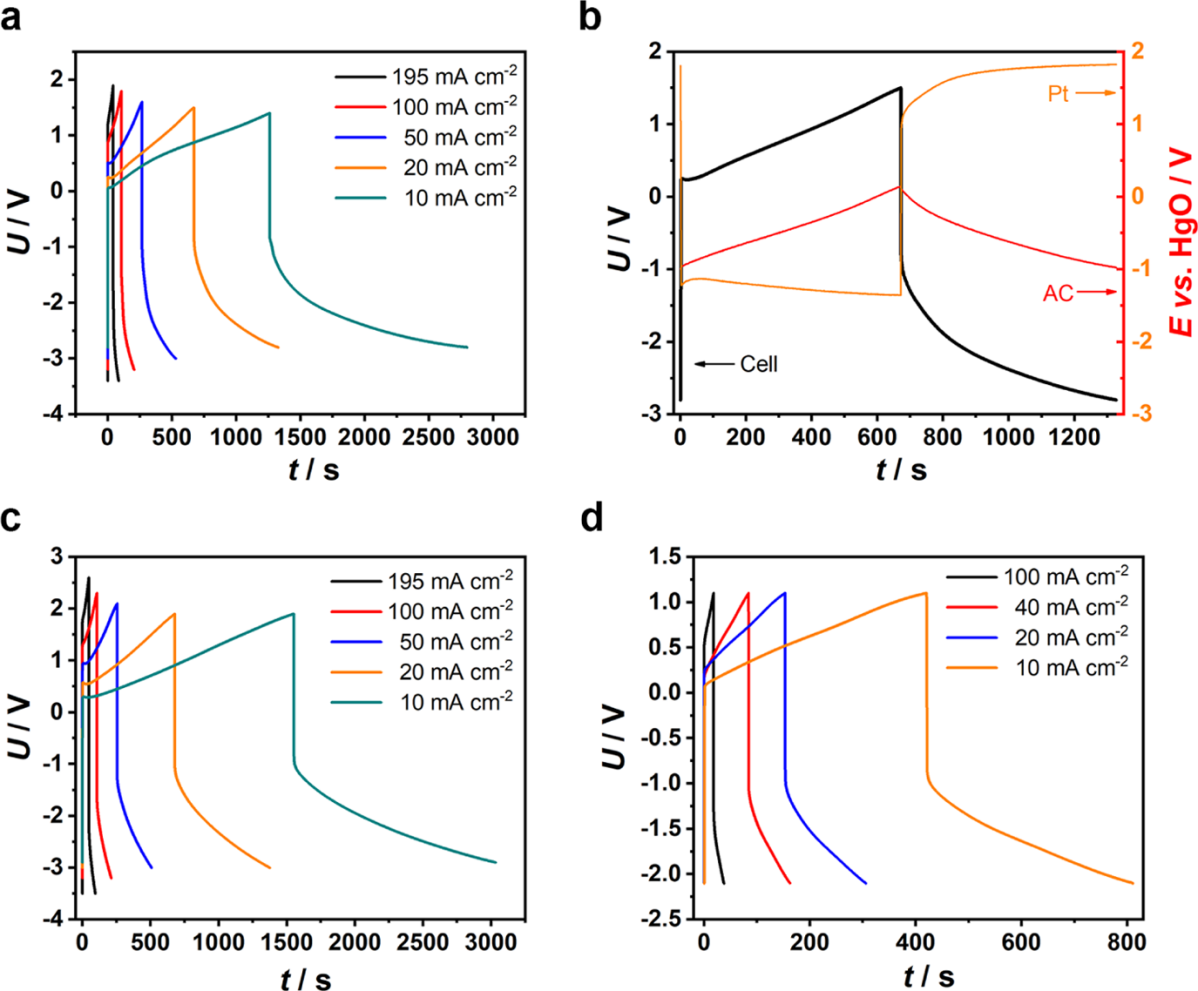
Electrochemical analyses. (a,c,d) Galvanostatic electrolysis curves of the half-electrolysis cell with AC and Pt wire electrodes in aqueous electrolytes of (a) 0.5 mol L^–1^ KOH + 0.5 mol L^–1^ NaCl, (c) 1.0 mol L^–1^ Na_2_SO_4_, and (d) 1.0 mol L^–1^ H_2_SO_4_ at indicated current densities. (b) Separately and simultaneously measured voltage–time curve of the half-electrolysis cell and potential–time curves of AC and Pt wire electrodes in 0.5 mol L^–1^ KOH + 0.5 mol L^–1^ NaCl at 20 mA cm^–2^.

In this work, all current densities were referred to the nominal area of the Pt electrode, while the AC electrode was always the working electrode for control purposes. In order to avoid the undesirable ClER, alkaline salt water (0.5 mol L^–1^ NaCl + 0.5 mol L^–1^ KOH) was used to simulate seawater with an alkaline additive as the electrolyte.^28^ The capacitance of the AC electrode was 16.6 F (Figure S1). Figure 1a shows the plot of cell voltage against the time of half-electrolysis in the galvanostatic mode. When the AC electrode began charging positively, the Pt electrode was a cathode and hence triggered HER. At 10.0, 20.0, 50.0, 100, and 195 mA cm^–2^, the voltage of the half-electrolysis for HER (*U*_HER_) reached initially 0.063, 0.256, 0.509, 0.868, and 1.166 V, respectively, which increased with the current density. At 10.0 and 20.0 mA cm^–2^, the reverse voltages of the half-electrolysis for OER (*U*_OER_) were initially −0.897 and −0.913 V, respectively.

It is interesting to note that the equivalent initial overall cell voltages (*U*_HER_ – *U*_OER_) of the half-electrolysis were only 0.960 and 1.169 V at 10.0 and 20.0 mA cm^–2^, smaller than the theoretical 1.229 V for electrolysis of water. However, this finding should not be seen as a deviation from thermodynamics. It agrees actually with the initial kinetic step of the HER and OER being electroadsorption, which could be more pronounced at lower current densities and lower initial *U*_OER_ in that the OER on the Pt electrode started against a positively charged AC electrode. Apparently, at higher current densities (Figures 1b, S2, and S3), the overpotential of the Pt electrode increased significantly, overshadowing the effect of the positively charged AC electrodes.

A HgO reference electrode was employed in the half-electrolysis cell to monitor changes of potential of the AC (*E*_AC_) and Pt (*E*_Pt_) electrodes at 20 mA cm^–2^, as shown in Figure 1b. When the cell was charged to 1.500 V (*U*_HER_), *E*_AC_ and *E*_Pt_ were 0.146 and −1.354 V, respectively. When discharging to −2.800 V (*U*_OER_), *E*_AC_ and *E*_Pt_ were −0.974 and 1.826 V, respectively. In both cases, the agreement between *U*_HER_ (or *U*_OER_) and the difference |*E*_AC_ – *E*_Pt_| indicates insignificant influence of the cell resistance. Upon switching on the current, the *U*_HER_ jump occurred, followed by a small peak, and then a linear increase in the remaining electrolysis time. This *U*_HER_ jump was accompanied by the opposite behavior for the initial *E*_Pt_, indicating a transient polarization in the HER. At the same time, *E*_AC_ varied linearly, indicating little or no polarization as expected for the supercapacitor electrode at relatively small applied current densities. A gradual transition without any peak can be seen on both the *U*_OER_ and *E*_Pt_ curves when the current was reversed, which is expected from the sluggish kinetics of OER. On the whole, the potential variation of the AC electrode was well followed by the cell voltage, implying that the potentials of the Pt electrode for both HER and OER should have been constant, which agreed with Figure 1b. When the current polarity was reversed from 20 to −20 mA cm^–2^, the cell voltage responded by a sharp fall, while *E*_Pt_ increased vertically. The changes in both the cell voltage and *E*_Pt_ were the same ca. 2.26 V, meaning that the large voltage change of the half-electrolysis cell was caused by the potential change of the Pt electrode when altering its reaction from HER to OER. Note that the *E*_AC_ curve shows no abrupt drop, which agrees with the insignificant ohmic loss across the cell at a small current as 20 mA cm^–2^. This means that the potential jump on the Pt electrode was caused by HER- and OER-related polarizations. At larger currents of 50 and 195 mA cm^–2^, the *E*_AC_ and *E*_Pt_ curves were similar in shape to their lower current counterparts as shown in Figures S2 and S3, although the ohmic loss became more apparent.

In order to rule out the possible electrode reaction of Cl^–^, chlorine-free solutions of 1.0 mol L^–1^ Na_2_SO_4_ and 1.0 mol L^–1^ H_2_SO_4_ were applied as the electrolytes, respectively. Similar electrolysis curves were obtained in Figure 1c,d. The increase and decrease in initial (*U*_HER_ – *U*_OER_) values are due to the fact that both HER and OER are kinetically unfavored in the neutral and favored in the acidic electrolyte. Saturated calomel electrodes were employed as the references to monitor the potential changes of the AC and Pt electrodes in the two electrolytes (Figures S4–S7).

Disregarding the nature of the electrolyte, the *E*_AC_ ranges in the half-electrolysis cells were always comparable with those in the three-electrode cells for capacitance measurement. The electrolysis time is determined by the capacitance of the AC electrode. Therefore, the overall electrolysis time of one course in the acidic electrolytes was the shortest among those in the three electrolytes because the capacitance of the AC electrode used in the acidic electrolyte was the smallest.

**Figure 2:**
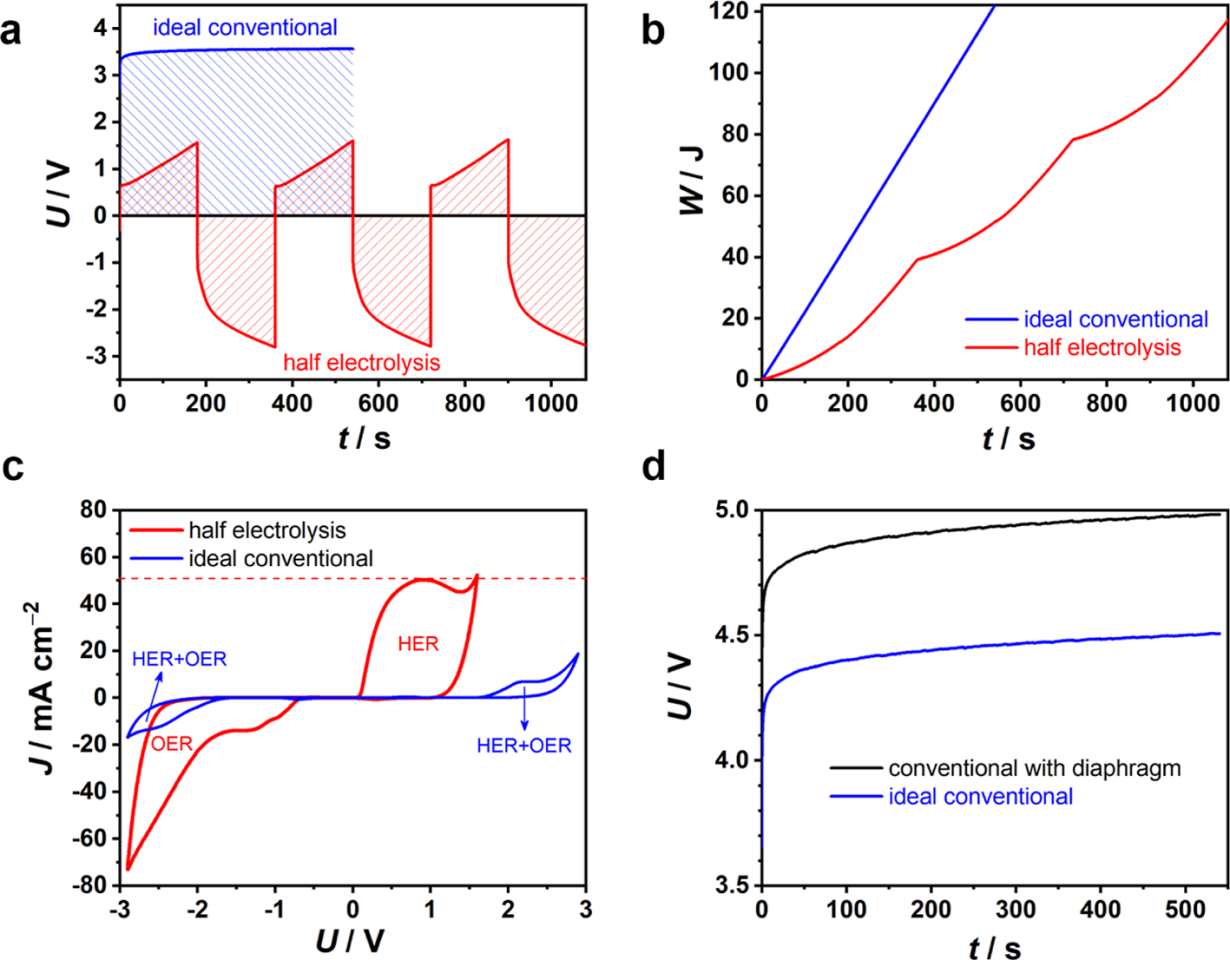
Electrochemical comparison between half-electrolysis and conventional electrolysis in an alkaline salt water electrolyte. (a) Galvanostatic electrolysis curves, (b) energy comparison at 50.0 mA cm^–2^, and (c) CVs at 5 mV s^–1^ of half-electrolysis (red line and texts) and ideal conventional electrolysis with two identical Pt wire electrodes without the diaphragm (blue line and texts) in alkaline salt water. (d) Galvanostatic electrolysis curves of a conventional cell with two identical Pt wire electrodes with or without the diaphragm at 50.0 mA cm^–2^.

It was then decided to compare the half-electrolysis cell with an ideal conventional electrolysis cell without the membrane but with two identical Pt electrodes in alkaline salt water at 50.0 mA cm^–2^ (Figure S8). In Figure 2a, the conventional cell responded with a constant voltage of ca. 3.548 V. Nevertheless, the voltage–time profile of the half-electrolysis cell is superimposed. Its *U*_HER_ values at all times were much lower than the voltages of the conventional cell, but this energy saving was consumed after reversed OER. To produce equal amounts of H_2_ and O_2_, the energy consumption of the half-electrolysis cell for three cycles was calculated to be 117 J, while that of the conventional cell was 122 J for a half-time of the half-electrolysis (Figure 2b). It means that the energy consumption of the half-electrolysis cell accounted for 96% of that of the ideal conventional cell. Similar results were obtained at 20.0 mA cm^–2^ for longer time (Figure S9).

The slightly lower energy consumption of the half-electrolysis cell agreed with the potential difference of 2.483 V for the Pt electrode in the half-electrolysis cell between the HER and OER branches within 0.35 s (Figure S2), smaller than 3.183 V at 0.35 s in the conventional cell (Figure 2a). This difference indicates that the Pt cathode (or anode) in the conventional cell experienced a higher polarization for HER (or OER). In the conventional cell, soon after the electrolysis started, the OER (or HER) at the Pt anode (or the cathode) would lead to a high H^+^ (or OH^–^) activity and hence a positive (or negative) concentration polarization.^29^ On the contrary, in the half-electrolysis cell, because the current was reversed alternatively, the Pt electrode always started HER (or OER) at a high activity of H^+^ (or OH^–^) after a given time of OER (or HER). Therefore, depolarization occurred on the Pt electrode upon switching the current polarity alternatively in half-electrolysis. However, in this work, because the ratio of the electrolyte volume to the Pt electrode area was large, the pH of the bulk electrolyte remained unchanged during electrolysis, meaning that the concentration polarization would saturate very quickly.

Figure 2c shows the cyclic voltammograms (CVs) of the half-electrolysis and ideal conventional electrolysis cells containing alkaline salt water. The voltage windows of the half-electrolysis for HER and OER were selected in line with those in Figure 1a at 50.0 mA cm^–2^. It is noted that the maximum currents of HER (52.4 mA cm^–2^) and OER (−73.0 mA cm^–2^) were too small to saturate (i.e., fully charge) and hence invoke faradaic reactions on the AC electrode on the CV time scale. In other words, the CV of the half-electrolysis cell reflected mainly the HER or OER features on the Pt electrode. The current onsets (at 1 mA cm^–2^) can be seen at 0.087 V (*U*_HER_) and −0.717 V (*U*_OER_). After the fast rise, the HER current passed through a peak and then to another increase. These two features agree with the HER going through electroadsorption, followed by H_2_ evolution. On the OER branch, the current response also indicated electroadsorption, followed by O_2_ evolution, but the currents of electroadsorption between −0.717 and −1.688 V were more sluggish and smaller than the HER counterparts due to the slow kinetics of OER.^30^ The symmetrical ideal conventional electrolysis cell gave rise to a CV of rotational symmetry of order of 2, which presented much smaller currents at the same scan rate. Unlike the CV of the half-electrolysis cell that exhibited features of HER or OER only in the positive or negative voltage scans, respectively, the CV of the conventional cell showed dominantly the OER features because the current flowing through the cell was controlled by the slow OER kinetics.

One of the expected advantages of half-electrolysis is the omission of the diaphragm and hence lower cost and energy consumption for water electrolysis. Figure 2d shows the voltage–time profiles of the alkaline salt water recorded at 50.0 mA cm^–2^ in a home-made conventional electrolysis cell with and without an asbestos diaphragm (Figure S10). The cell without the diaphragm expressed a nearly constant voltage of ca. 4.459 V, while that with a diaphragm expressed ca. 4.943 V, reflecting the effect of the diaphragm resistance of 7.563 Ω, which was close to 7.060 Ω calculated according to electrochemical impedance spectroscopy (EIS) results in Figure S11. The corresponding energy consumptions were 154 and 170 J in the absence and presence of the diaphragm, respectively, after electrolysis for 540 s. The increase in energy consumption by 10% resulting from the diaphragm was not unexpected and could be worse at a higher current density. In comparison with the conventional cell with the diaphragm, the half-electrolysis cell under the same conditions saved about 14% in energy input. The same 14% energy could be saved at 20.0 mA cm^–2^ (Figure S12).

In the case of conventional electrolysis of 1 mol L^–1^ Na_2_SO_4_, a PEM was used to separate the H_2_ and O_2_ chambers. According to Figures S13 and S14, half-electrolysis saved 8% of energy input. After a few conventional electrolysis experiments, PEM was distorted mainly due to the swelling of PEM (Figure S15).

**Figure 3:**
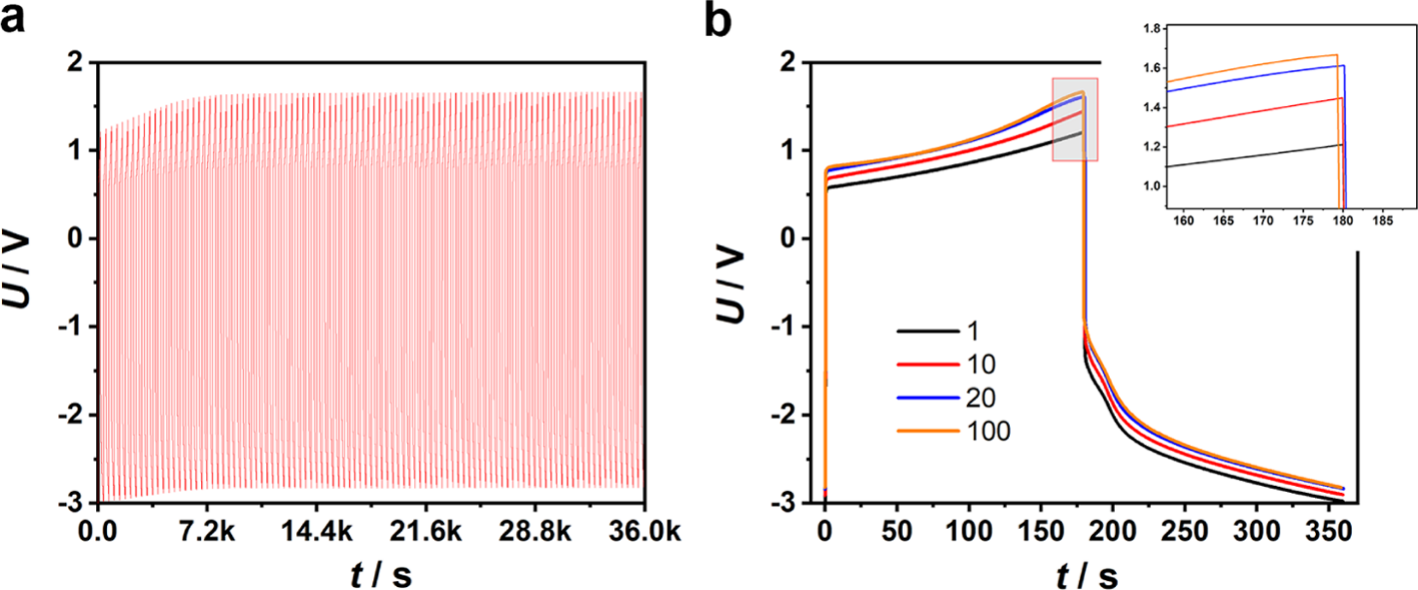
Cyclic performance of the half-electrolysis cell containing alkaline salt water at 50 mA cm^–2^. (a) Galvanostatic electrolysis curve recorded continuously over 10 h (100 cycles). (b) Superimposed electrolysis curves of the 1st, 10th, 20th, and 100th cycles.

In continuous operation, both the anode and cathode of a conventional electrolysis cell will experience catalyst aging^31^ and hence need to be replaced. In the half-electrolysis cell, the AC electrode is capable of tens of thousands of cycles,^32−34^ and thus only the electrolysis electrode loaded with the catalysts needs replacement, which can be translated into significant cost saving. The cyclic performance of the half-electrolysis cell containing alkaline salt water was tested at 50.0 mA cm^–2^ for 180 × 2 s over 100 cycles, as shown in Figure 3a. It can be seen that the electrolysis curve rose continuously until the 20th cycle. Since then, each cycle of the electrolysis curve nearly overlaid, suggesting high electrochemical stability of both the Pt and AC electrodes (Figure 3b). Because the nonlinear shape of the electrolysis curve was determined by mostly OER and also HER to a lesser degree, the upward shift of the initial cycles of the electrolysis curve should have resulted from changes on the AC electrode. A plausible explanation is that the charge balancing ions, upon repeated ingression and egression, gradually rearranged themselves inside the pores of the AC electrode.

In order to check whether ClER occurred along with OER in the half-electrolysis of alkaline salt water, another 50 cycles after the first 100 cycles were used to collect the produced gas. The gas chromatogram-mass spectroscopy (GC-MS) result in Figure S16 revealed that no Cl_2_ gas was produced, which could be attributed to high pH of the electrolyte.^35^

The advantages in the cyclic performance of the half-electrolysis were also revealed in 1.0 mol L^–1^ Na_2_SO_4_ and H_2_SO_4_, shown in Figures S17–S22.

**Figure 4:**
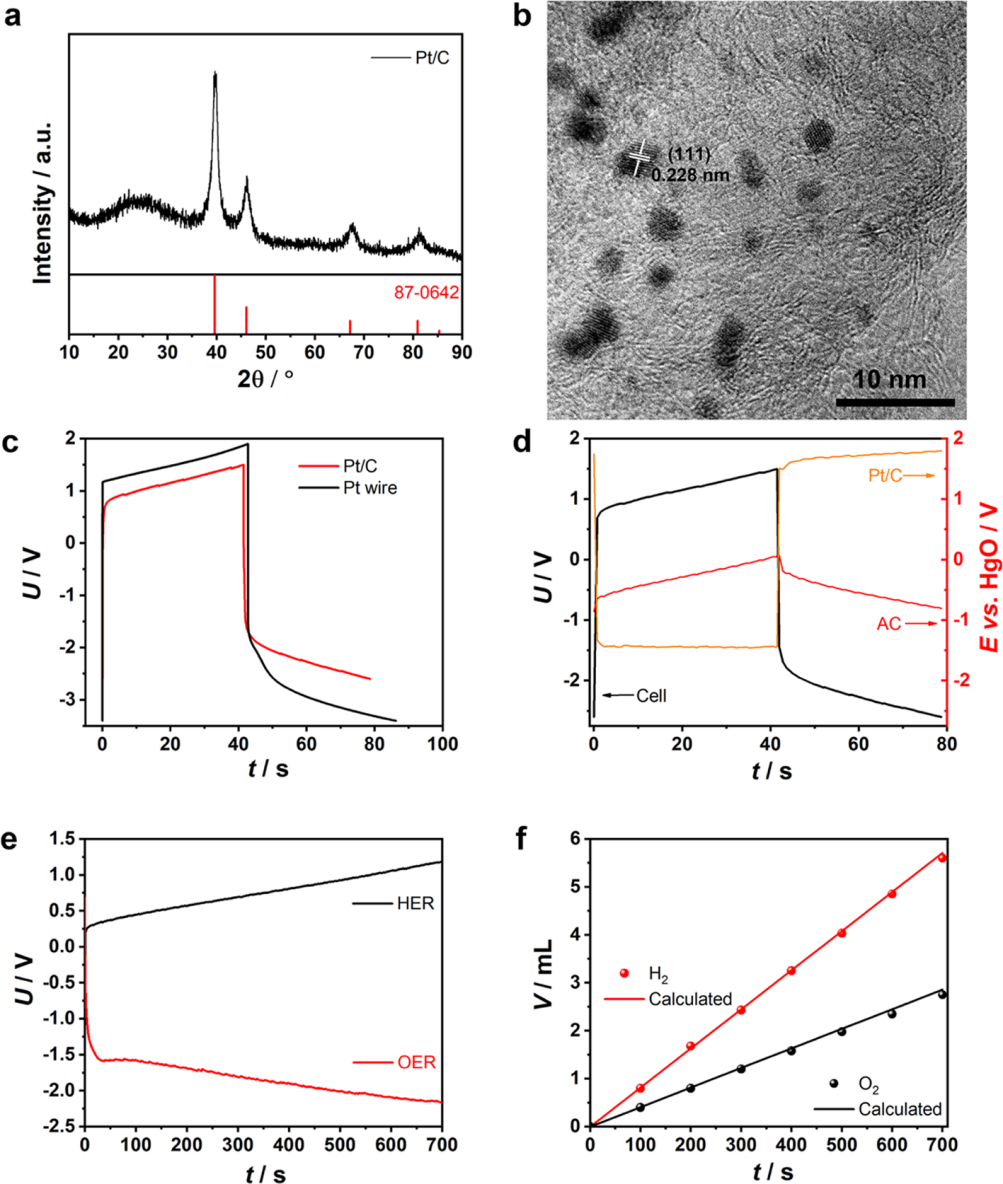
Half-electrolysis with the Pt/C electrode. (a) XRD pattern and (b) TEM image of Pt/C. (c) Half-electrolysis curves with Pt/C and Pt wire electrodes at 195 mA cm^–2^. (d) Voltage–time curve of the half-electrolysis cell, potential–time curves of the AC and Pt/C electrodes at 195 mA cm^–2^ in alkaline salt water. (e) Galvanostatic electrolysis curves of half-electrolysis of alkaline salt water consisting of the AC electrode of 54.1 F in capacitance and Pt/C pasted on the Pt wire electrode at 50 mA cm^–2^. (f) Volumes of H_2_ (red dots) and O_2_ (black dots) collected with burette (markers) at different times and calculated (lines) from the charge passed during electrolysis.

Considering that the Pt wire electrode had a low active specific surface, Pt/C coated on the Pt wire with more active sites was also applied in electrolysis.^36^ The XRD peaks of the Pt/C material in Figure 4a were consistent with those of Pt (JCPDF #87-0642). Figures 4b and S23 show the Pt nanoparticles with an average size of ca. 2 nm on AC, in which the planar distance of 0.228 nm corresponded to Pt(111). Figure S24 shows the *IR*-corrected voltammetry curves of Pt/C and Pt wire. The Pt/C coating exhibited evidently lower onset potentials and much smaller overpotentials than the Pt wire at the same current density, especially during HER and even OER. The Tafel slope of Pt/C during HER was half of that of the Pt wire. However, Pt/C had a very large Tafel slope during OER. One factor should be considered that Pt/C with 1.5 mol % Pt was not a very active catalyst for OER; the other factor would be discussed later after the DPD method for the Pt wire and Pt/C. In Figure 4c, with a similar electrolysis time at the same 195 mA cm^–2^, the cell with Pt/C showed a voltage range from −2.600 to 1.500 V, which was evidently smaller than that from −3.400 to 1.900 V for that with the Pt wire, suggesting that Pt/C had better, especially OER, catalytic activity than the Pt wire. Unlike the Pt wire electrode with a shoulder in the initial OER curve, the Pt/C electrode presented an intact straight line. Figure S25 shows the EIS spectra of the half-electrolysis cells consisting of the Pt wire and Pt/C, giving rise to the equivalent series resistance of 2.222 and 2.708 Ω, respectively. It was worth noting that the charge-transfer resistance of Pt/C was much higher than that of the Pt wire due to the porous structure in Pt/C in which both electronic and ionic charge-transfer kinetics occurred.

*E*_Pt/C_ were monitored by a HgO reference electrode in the half-electrolysis cell at 195 mA cm^–2^ (Figure 4d), showing that the *E*_Pt/C_ values during HER and OER were ca. −1.443 and 1.726 V, respectively. Contrastively, inFigure S3, the *E*_Pt_ values during HER and OER were ca. −1.846 and 2.390 V, respectively, indicating much larger polarizations of the Pt wire, especially for OER. The shoulder in the initial *U*_OER_ curve in the half-electrolysis cell could have resulted from the Pt wire electrode (Figure S3). In contrast, no shoulder can be seen in the *E*_Pt/C_ curve in Figure 4d.

In order to collect and analyze the gases produced in the half-electrolysis of the alkaline salt water, an AC electrode with 54.1 F was employed in a sealed half-electrolysis cell comprising the same Pt wire electrode at 50 mA cm^–2^. When HER and OER were each underway for 600 s, the evolved volumes of H_2_ and O_2_ were 4.7 and 1.6 mL (Figure S26), corresponding to the current efficiencies for HER and OER of 97.0 and 65.2%, respectively. The current efficiency of OER was much lower than that of conventional electrolysis of the pure KOH electrolyte,^37^ even if no Cl_2_ gas was produced.

As previously reported,^38^ the main competitive anodic reactions at high pH are the OER and hypochlorite formation. Hence, following OER, the alkaline salt water electrolyte was examined using the *N*,*N*-diethyl-*p*-phenylenediamine (DPD) reagent to determine whether any hypochlorite formed. Figure S27 shows a light pink color in the reagent, suggesting that ca. 0.1 mg L^–1^ hypochlorite was produced, which resulted in the low efficiency of OER.

When Pt/C replaced the Pt wire, after HER and OER each for 700 s, the corresponding volumes of H_2_ and O_2_ were 5.6 and 2.75 mL, corresponding to 98.0 and 96.3% in current efficiency, respectively (Figure 4e,f). The good current efficiency of OER suggests that Pt/C with a higher active surface than the Pt wire was effective to impede the hypochlorite formation. It is believed that better catalysts for OER with higher specific surface area can achieve an OER efficiency of nearly 100%. After the first and fourth electrolysis cycle on Pt/C, the DPD method featured no visible pink color in the reagents (Figure S28), suggesting that no hypochlorite formed during OER on Pt/C. The competitive side reaction of hypochlorite formation in the Pt wire also suggests that it is unfair to compare the OER Tafel slopes between Pt/C and the Pt wire. It can be expected that the commercial Pt/C materials, such as HiSPEC2000, 8100, and 9100 (Johnson Matthey) in which the maximum crystallite sizes of Pt are below 3 nm, have the same effect to suppress the side reactions in the half-electrolysis of simulated seawater. Additionally, it is demonstrated that the half-electrolysis of water with the aid of the AC electrode has obvious advantages that it can adapt to aqueous electrolytes with different pHs and afford a large current density and long lifetime. The comparisons of the half-electrolysis to the decoupling of water splitting with faradaic reaction electrodes are shown in Table S1, suggesting that our work first proved the energy saving for half-electrolysis of water.

## Conclusions

In summary, we have applied the half-electrolysis concept to water electrolysis, whose cell comprises one AC supercapacitor electrode and the other Pt wire electrolysis electrode in alkaline salt water (simulant of seawater), neutral Na_2_SO_4_, and acidic H_2_SO_4_ electrolytes. H_2_ and O_2_ can be stepwise produced on the same Pt electrolysis electrode, so a diaphragm is avoided in the cell design. When charging the supercapacitor electrode, HER occurs at the electrolysis electrode as a cathode, while discharging enables OER as an anode. The energy consumption of the half-electrolysis is smaller than that of the practical conventional electrolysis with a diaphragm. The half-electrolysis of water can prevent the gas permeation, reduce the cost of the whole instrument, increase the electrolysis rate, and further reduce the energy consumption of electrolysis, inferring a hopeful prospect to innovate the science and application of electrolysis of water.

## Supporting Information Available

Methods, electrochemical analysis on half-electrolysis in alkaline salt water, Na_2_SO_4_ and H_2_SO_4_ electrolytes, energy consumption comparison in alkaline salt water and Na_2_SO_4_ electrolytes, GC–MS to check Cl_2_ for half-electrolysis in alkaline salt water, cyclic performance of half-electrolysis in Na_2_SO_4_ and H_2_SO_4_ electrolytes, TEM images of Pt/C, voltammetry curves and Tafel slopes of Pt wire and Pt/C, EIS of the half-electrolysis cell consisting of Pt wire and Pt/C, analysis of gas and reactions in half-electrolysis with Pt wire and Pt/C in alkaline salt water, and comparison of half-electrolysis to decoupling of water splitting (PDF)
