# Supplementary material for: Half-Electrolysis of Water with the Aid of a Supercapacitor Electrode
Source: ACS Appl Energy Mater. 2023 May 19;6(11):6104–10. doi: 10.1021/acsaem.3c00615 (PMC10265655; doi:10.1021/acsaem.3c00615)
Supplement: Supplementary file 1 — ae3c00615_si_001.pdf [file ae3c00615_si_001.pdf]

Supporting Information

Half-Electrolysis of Water with the Aid of a Supercapacitor Electrode

Yao Chen[1] and George Zheng Chen[2,⋆]

[1] The State Key Laboratory of Refractories and Metallurgy, College of Materials and Metallurgy, Wuhan University of Science and Technology, Wuhan 430081, P. R. China

[2] Department of Chemical and Environmental Engineering, Faculty of Engineering, University of Nottingham, Nottingham NG2 7RD, UK

Corresponding author. E-mail: george.chen@nottingham.ac.uk (G. Z. Chen).

Methods

The commercial AC was from Hua County AC Factory, China. The commercial Pt wire electrode (Φ0.2×2 cm^2) has a nominal surface area of ca. 1.28 cm^2.

Pt/C was prepared by a facile precipitation method. Typically, 0.08 g commercial AC was dispersed into 2 mL water and then mixed with 0.32 mol L^{-1}, 0.32 mL of K_2PtCl_4 aqueous solution. 0.04 g $NaBH_4$ with 0.01 g NaOH in 0.5 mL water was injected into the mixed solution and stirred for 30 min. The sample was collected after centrifugation, washing and desiccation under vacuum.

The X-ray diffraction (XRD) patterns were measured by XPert PRO MPD with Cu-Kα radiation (30 kV, 30 mA). The transmission electron microscopy (TEM) images were taken by Transmission electron microscope (TEM, JEM-2100UHR). The generated gas was analyzed by gas chromatography-mass spectroscopy (GC-MS) with QP2010 plus, Shimadzu. It is very challenging to measure the XRD pattern of the single Pt wire (electrode), but the XRD pattern of crystalline or powdery Pt is available from the literature.[31]

For the half-electrolysis of water, a Pt wire or Pt/C coating on the Pt wire with the same nominated surface area was used as the electrolysis electrode. The supercapacitor electrode was consisting of the commercial AC, acetylene black and polyvinylidene difluoride. The distance between the two electrodes was 1.6 cm. If the three electrode cells were carried out, a HgO electrode or saturated calomel electrode (SCE) was used as the reference electrode. These electrodes were inserted into 70 mL of aqueous electrolyte of (0.5 mol L^{-1} KOH + 0.5 mol L^{-1} NaCl), 1.0 mol L^{-1} Na_2SO_4, or 1.0 mol

L^{-1} H_2SO_4 in a cylindrical cell. The electrolyte of (0.5 mol L^{-1} KOH + 0.5 mol L^{-1} NaCl) in Fig. 1a was selected because the lowest additive of KOH in simulate seawater was 0.5 mol L^{-1} to prevent the evolution of Cl_2. The other two were selected because they are conventionally used, especially for supercapacitor electrode, due to the balance of the conductivity and viscosity and optimal capacitive performance.[S1] In order to compare the energy consumptions between the half-electrolysis and conventional electrolysis, the same cylindrical cell with the same 70 mL electrolyte was employed except for the two identical Pt wire electrodes with the same electrode distance were used as the electrolysis electrodes, for example for **Fig. 2a** in the main manuscript.

In order to compare the energy consumptions of the conventional electrolysis with to without the diaphragm or membrane, the two identical Pt wire electrodes were applied in the same home-made cuboid cell with the same 50 mL electrolyte, for example for **Fig. 2d** in the main manuscript. The distance between the two Pt wire electrodes was 3.1 cm without diaphragm. The thickness of the asbestos diaphragm was 0.36 cm while the thickness of PEM could be ignored.

The electrochemical measurements were conducted on a CHI660E electrochemical workstation and LAND CT2001A system, during which the supercapacitor electrodes were employed as the working electrode. The galvanostatic electrolysis curves of half-electrolysis cells were collected in the potential priority current switching mode. But the comparing the energy consumption of the half-electrolysis to conventional electrolysis and cyclic performance of the half-electrolysis were measured in the time priority current switching galvanostatic mode unless noted

otherwise.

For the gas collection, a gas burette filled with water was connected to the sealed half-electrolysis cell containing (0.5 mol L^{-1} KOH + 0.5 mol L^{-1} NaCl). The volume of the evolved gas was monitored by recording the displacement of water in the burette. The corresponding potential range of the AC electrode was strictly controlled within −0.9 to 0.2 V *vs*. HgO during both the HER and OER processes to avoid invoking any possible side reaction on the AC electrode, hence ensuring the purity of H_2 or O_2 produced on the Pt wire electrode.

Electrochemical analysis on half-electrolysis in alkaline salt water electrolyte.

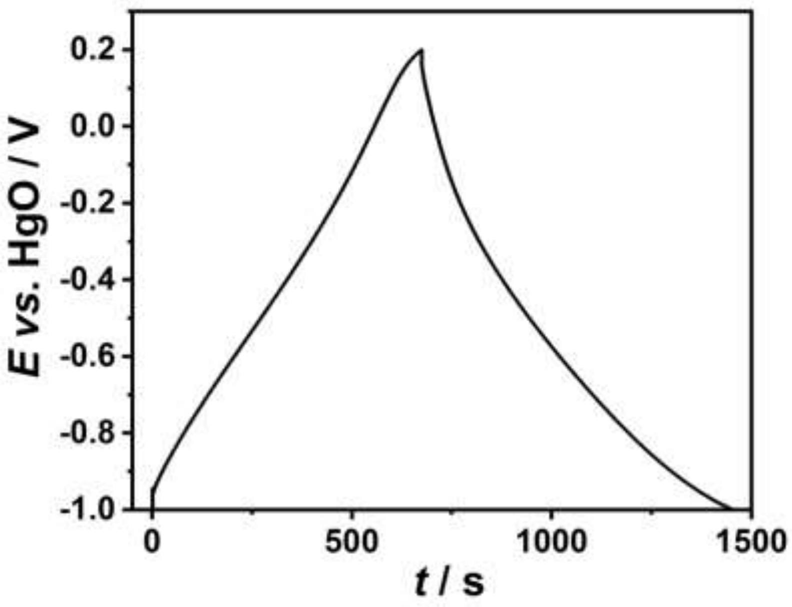

Fig. S1 | GCD curve of commercial AC in (0.5 mol L^{-1} KOH + 0.5 mol L^{-1} NaCl) electrolyte at 20.0 mA cm^{-2} (against electrolysis electrode as default).

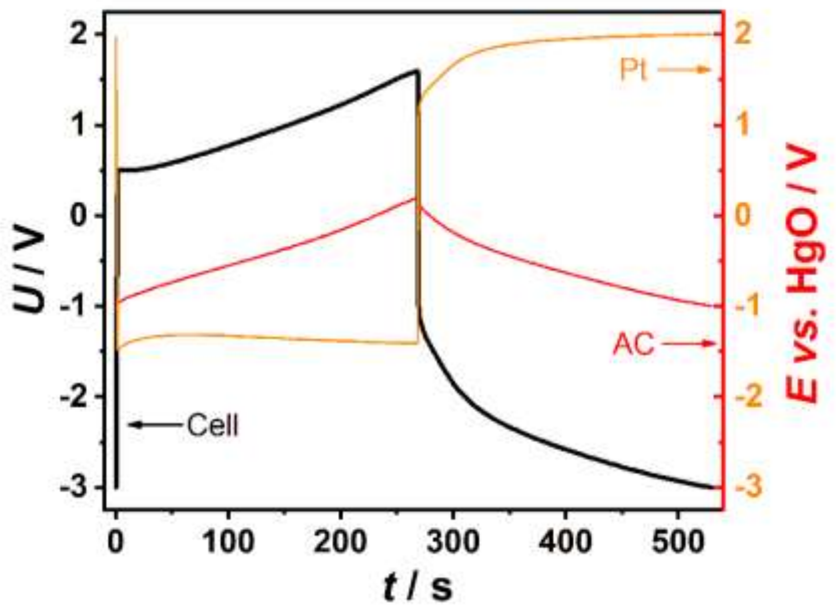

Fig. S2 | Separately and simultaneously measured voltage-time curve of half-electrolysis cell, potential-time curves of AC and Pt wire electrodes in (0.5 mol L^{-1} KOH + 0.5 mol L^{-1} NaCl) electrolyte at 50.0 mA cm^{-2}.

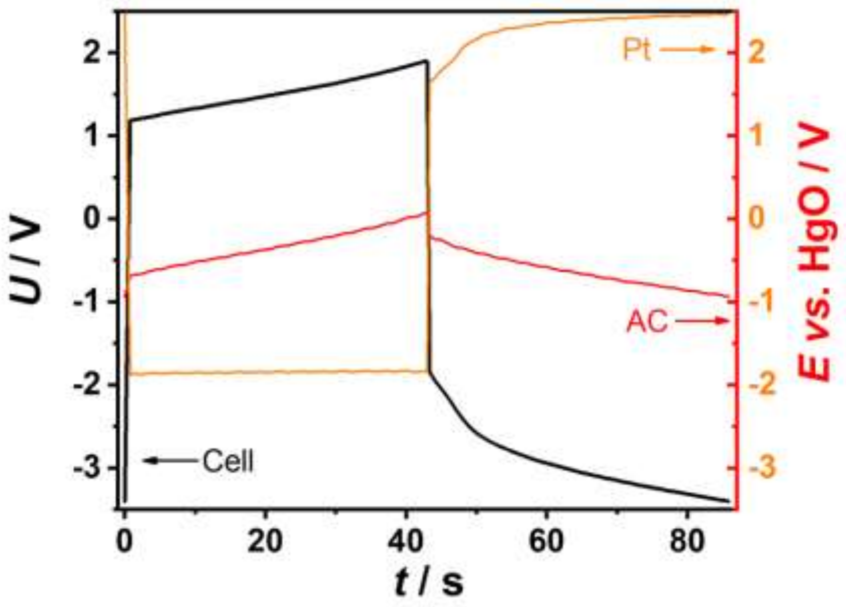

Fig. S3 | Separately and simultaneously measured voltage-time curve of half-electrolysis cell, potential-time curves of AC and Pt wire electrodes in (0.5 mol L^{-1} KOH + 0.5 mol L^{-1} NaCl) electrolyte at 195 mA cm^{-2}.

Electrochemical analysis on half-electrolysis in Na_2SO_4 electrolyte.

Another AC electrode of 11.8 F in capacitance was used as the supercapacitor electrode (**Fig. S4**). **Fig. 1**c plots the galvanostatic curves of the half-electrolysis at different current densities. It can be seen that at 10.0, 20.0, 50.0, 100 and 195 mA cm^{-2}, the initial U_{HER} values were 0.302, 0.576, 0.944, 1.274 and 1.724 V respectively. For OER, at 10.0 and 20.0 mA cm^{-2}, the initial U_{OER} reached −0.968 and −1.163 V respectively. Note that the initial (U_{HER} – U_{OER}) value also increased with the current density, but were all larger than those obtained in the alkaline salt electrolyte at the same current densities. This change is likely due to both HER and OER are kinetically unfavoured in a neutral electrolyte.

A saturated calomel electrode (SCE) was employed as the reference to monitor the potential changes of the AC and Pt electrodes, as shown in **Fig. S5**. When the cell was charged to 1.900 V (U_{HER}) at 20.0 mA cm^{-2}, E_{AC} and E_{Pt} were 0.626 and −1.274 V, respectively. When discharging to −3.000 V (U_{OER}), E_{AC} and E_{Pt} were −0.876 and 2.124 V, respectively. It can be seen the initial small peak also appeared on the U_{HER} branch of the electrolysis curve.

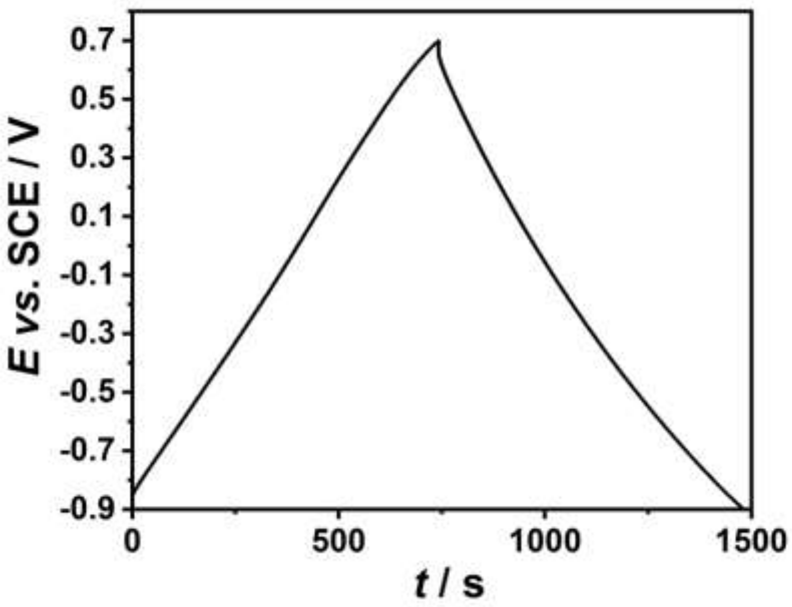

Fig. S4 | GCD curve of commercial AC in 1.0 mol L^{-1} Na_2SO_4 electrolyte at 20.0 mA cm^{-2}.

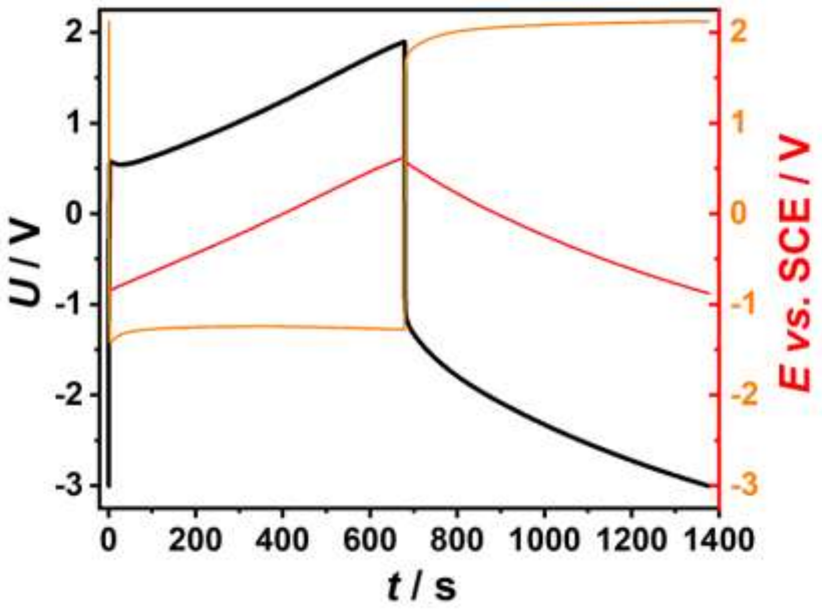

Fig. S5 | Separately and simultaneously measured voltage-time curve of half-electrolysis cell, potential-time curves of AC and Pt wire electrodes in 1.0 mol L^{-1} Na_2SO_4 electrolyte at 20.0 mA cm^{-2}.

Electrochemical analysis on half-electrolysis in H_2SO_4 electrolyte.

Considering that Pt is most active in an acidic electrolyte for HER, 1.0 mol L^{-1} H_2SO_4 was tested in the half-electrolysis cell as well. The AC electrode used in the acidic electrolyte had a capacitance of 4.6 F (**Fig. S6**). This time, upon trails, the voltage range for the galvanostatic electrolysis was set between −2.100 and 1.100 V. In **Fig. 1**d, it can be seen that at 10.0, 20.0, 40.0 and 100 mA cm^{-2}, the initial U_{HER} reached 0.072, 0.189, 0.201 and 0.508 V, respectively. At 10.0 and 20.0 mA cm^{-2}, the initial U_{OER} were −0.955 and −1.072 V, respectively. It is interesting to note that in this acidic electrolyte, the initial (U_{HER} – U_{OER}) values at > 20.0 mA cm^{-2} were the smallest among those in the three electrolytes, benefiting apparently from the promoted HER kinetics.

The SCE reference electrode was used to monitor the potential changes of the AC and Pt electrodes in the half-electrolysis cell again, as shown in **Fig. S7**. When the cell was charged to 1.100 V at 20 mA cm^{-2}, E_{AC} and E_{Pt} were 0.788 and −0.312 V, respectively. When discharging to −2.100 V, E_{AC} and E_{Pt} were −0.079 and 2.021 V, respectively. Interestingly, the small peak at the initial HER disappeared or became invisible in the H_2SO_4 electrolyte, which verifies the above attribution to the HER polarisation in alkaline and neutral electrolytes.

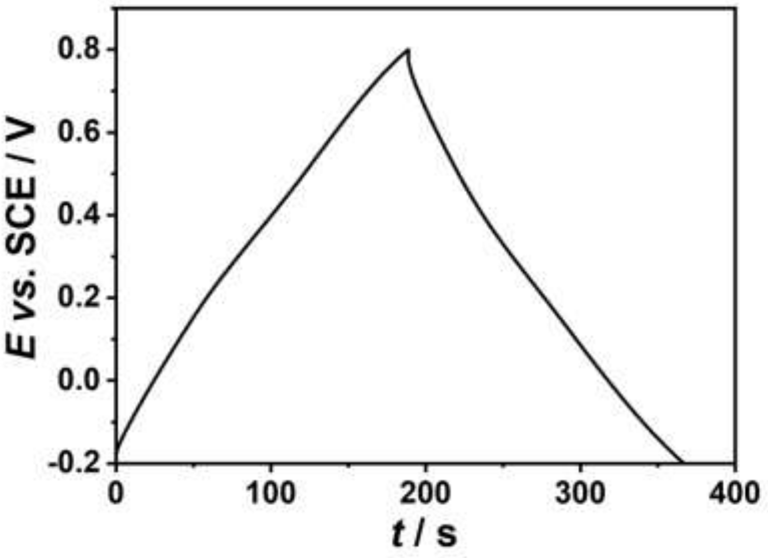

Fig. S6 | GCD curve of commercial AC in 1.0 mol L^{-1} H_2SO_4 electrolyte at 20.0 mA cm^{-2}.

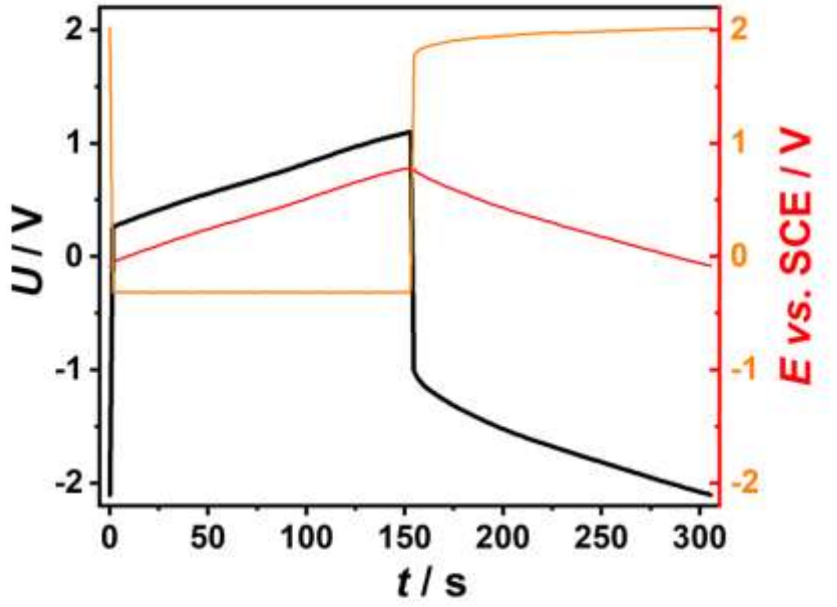

Fig. S7 | Separately and simultaneously measured voltage-time curve of half-electrolysis cell, potential-time curves of AC and Pt wire electrodes in 1.0 mol L^{-1} H_2SO_4 electrolyte at 20.0 mA cm^{-2}.

Energy consumption comparison between half and ideal conventional cell in alkaline salt water electrolyte.

In the alkaline salt water electrolyte, when the current density was changed to 20.0 mA cm^{-2}, the energy consumption was 110 J for 3 cycles of half-electrolysis, and 101 J for ideal conventional electrolysis upon producing the same amounts of H_2 and O_2 gases (**Fig. S9**). It meant that the energy consumption of the half-electrolysis cell accounted for 92% of that of the ideal conventional cell, which was slightly lower than 96% at 50.0 mA cm^{-2}.

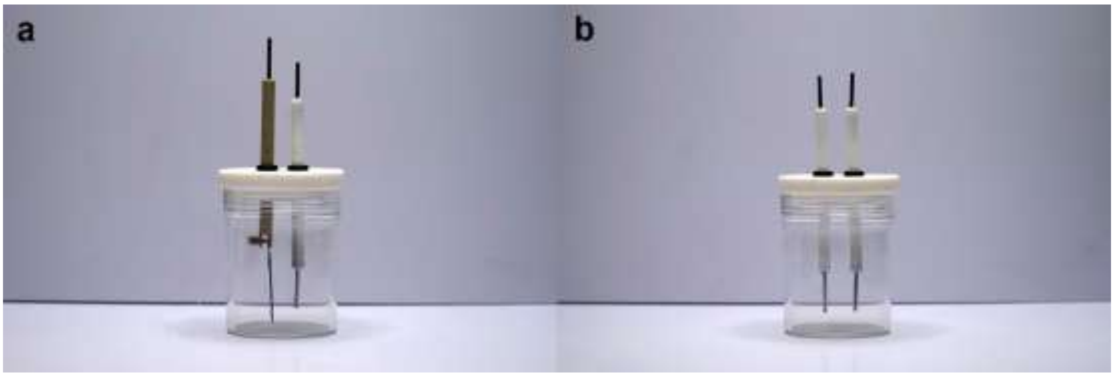

Fig. S8 | Digital photos of cylindrical cells of **a**, half and **b**, ideal conventional electrolysis with electrode distance of 1.6 cm.

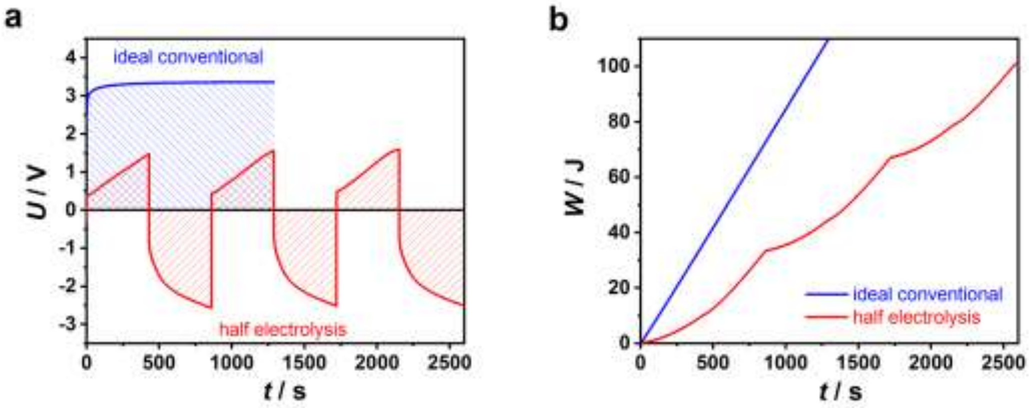

Fig. 9 | **a**, Galvanostatic electrolysis curves and **b**, energy consumption (*W*) of a half-electrolysis cell (red line) and a conventional electrolysis cell with two identical Pt wire electrodes without diaphragm (blue line) in (0.5 mol L^{-1} KOH + 0.5 mol L^{-1} NaCl) electrolyte at 20.0 mA cm^{-2}.

Energy consumption comparison between ideal conventional and conventional cell with diaphragm in alkaline salt water electrolyte.

The diaphragm was also used for electrolysis at 20.0 mA cm^{-2}. According to **Fig. S11**, the voltages of the conventional cell without and with diaphragm were *ca.* 3.569 and 3.806 V, corresponding to 118 and 125 J of energy input respectively after electrolysis for 1291 s. In comparison with the conventional cell with the diaphragm, the half-electrolysis cell under the same conditions saved 14% in energy input, which was the same as that at 50.0 mA cm^{-2}.

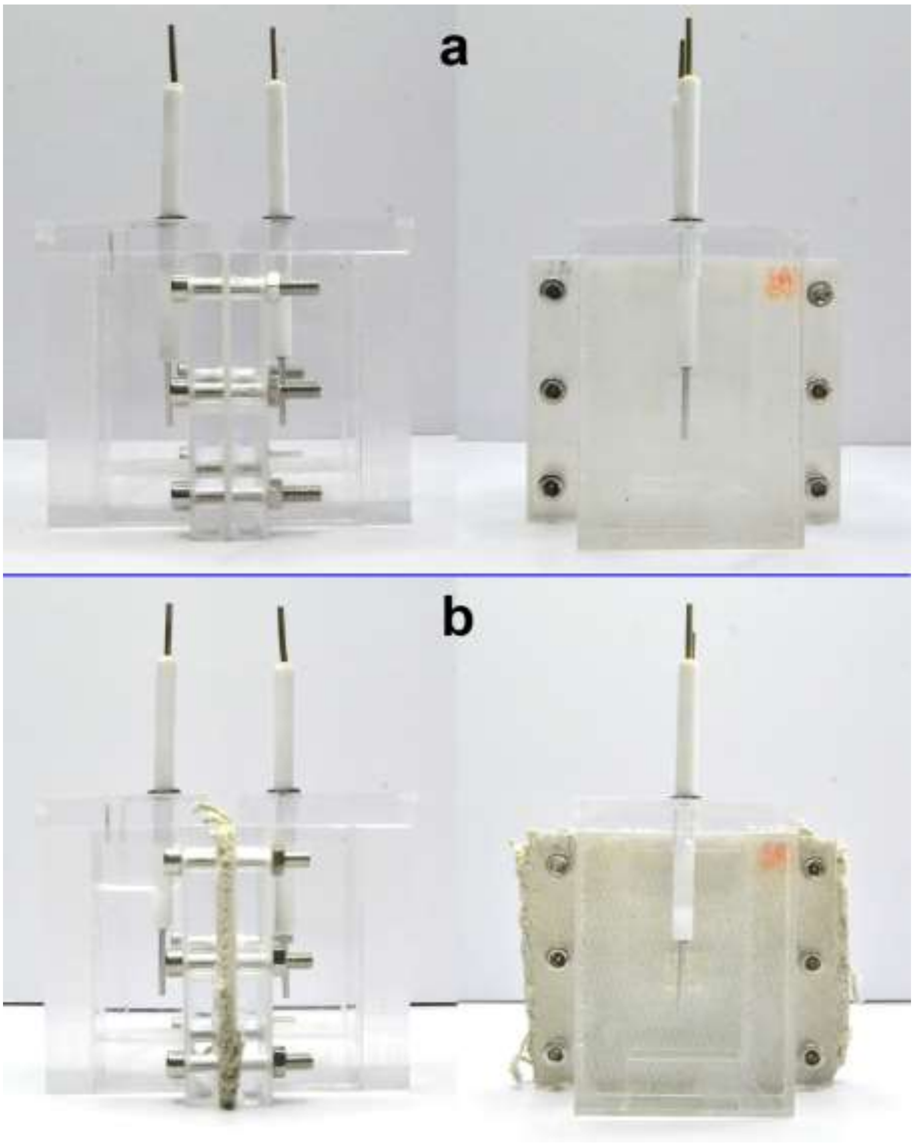

Fig. S10 | Digital photos of home-made cuboid conventional electrolysis cells **a**, with and **b**, without asbestos diaphragm. Electrolyte only filled into in left chamber in **b** to demonstrate that asbestos diaphragm can impede electrolyte penetration.

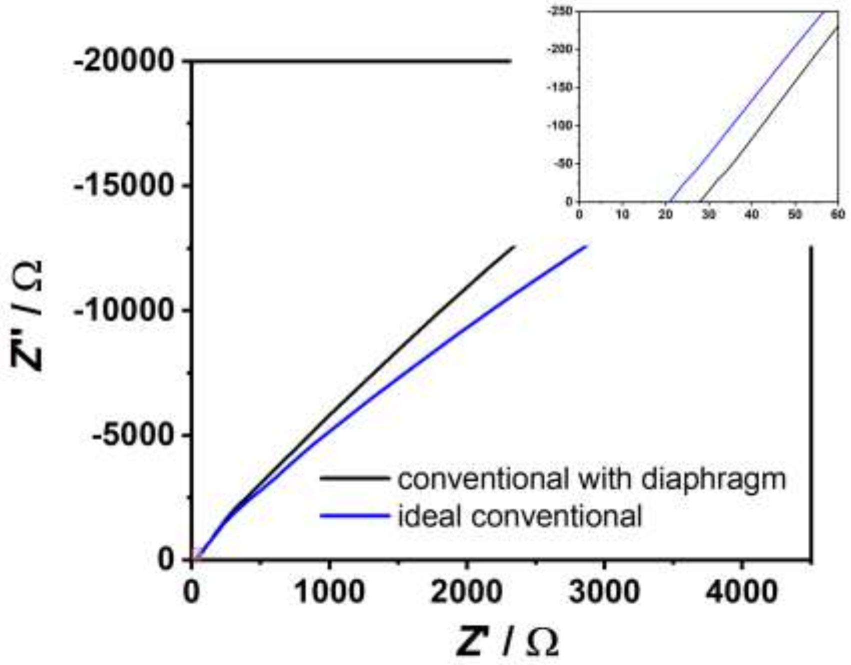

Fig. S11 | EIS spectra of conventional cell with two identical Pt wire electrodes with or without diaphragm in (0.5 mol L−1 KOH + 0.5 mol L−1 NaCl) electrolyte.

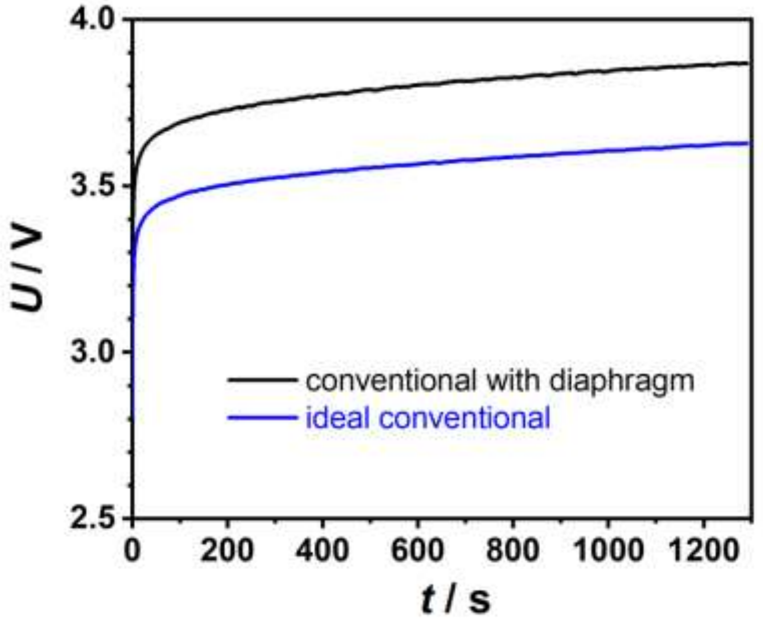

Fig. S12 | Galvanostatic electrolysis curves of conventional cell with two identical Pt wire electrodes with or without diaphragm in (0.5 mol L^{-1} KOH + 0.5 mol L^{-1} NaCl) electrolyte at 20.0 mA cm^{-2}.

Energy consumption comparison among the half-electrolysis, ideal conventional and conventional cell with PEM in Na_2SO_4 electrolyte.

In the neutral Na_2SO_4 electrolyte, at 50.0 mA cm^{-2}, the energy consumption was 187 J for 4 cycles of half-electrolysis, and 190 J for ideal conventional electrolysis upon producing the same amounts of H_2 and O_2 gases (**Fig. S12**). A PEM was used to separate the H_2 and O_2 chambers for electrolysis. According to **Fig. S13**, the voltages of the conventional cell without and with PEM were *ca.* 4.923 and 5.252 V, corresponding to 85 and 80 J of energy input respectively after electrolysis for 254 s. Under the same conditions, half-electrolysis saved 8% of energy input. It was observed in this work that the PEM was evidently distorted after a few conventional electrolysis experiments, mainly due to the swelling of PEM, as shown in **Fig. S14**.

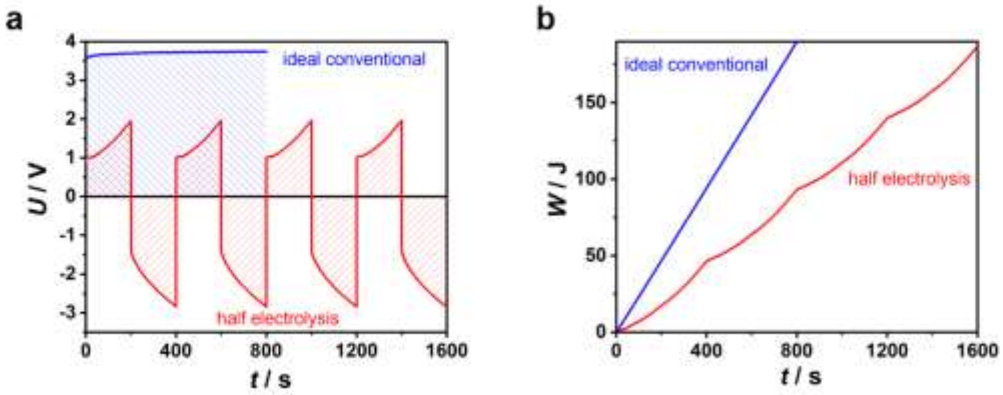

Fig. S13 | a, Galvanostatic electrolysis curves and **b**, energy consumption (*W*) of a half-electrolysis cell (red line) and a conventional electrolysis cell with two identical Pt wire electrodes without diaphragm (blue line) in 1.0 mol L^{-1} Na_2SO_4 electrolyte at 50.0 mA cm^{-2}. The total amount of charge, and hence the total volume of H_2 and O_2 gases produced in both cells were the same.

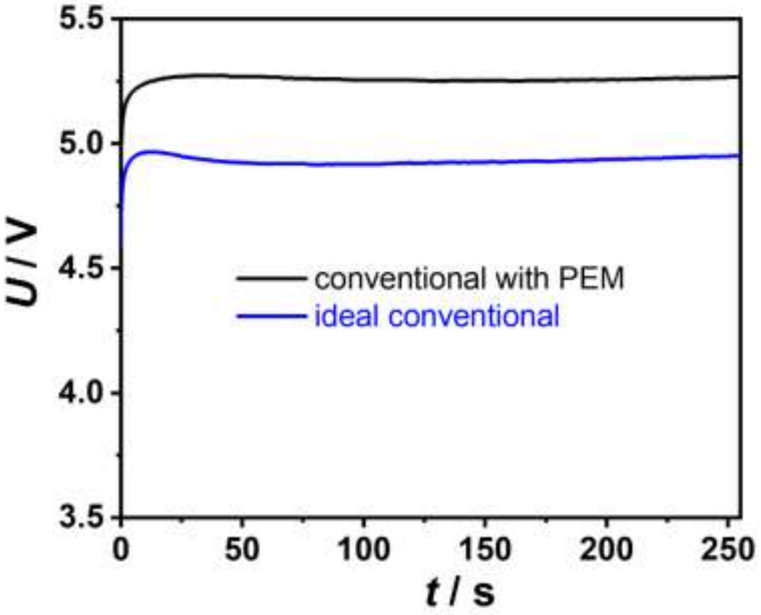

Fig. S14 | Galvanostatic electrolysis curves of conventional cell with two identical Pt wire electrodes with or without diaphragm in 1.0 mol L^{-1} Na_2SO_4 electrolyte at 50.0 mA cm^{-2}.

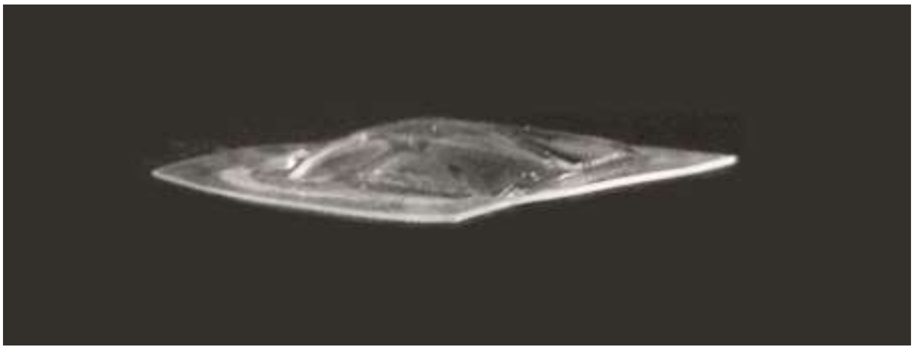

Fig. S15 | Digital photo of PEM after few conventional electrolysis in 1.0 mol L^{-1} Na_2SO_4 electrolyte.

Check Cl_2 for half-electrolysis in alkaline salt water.

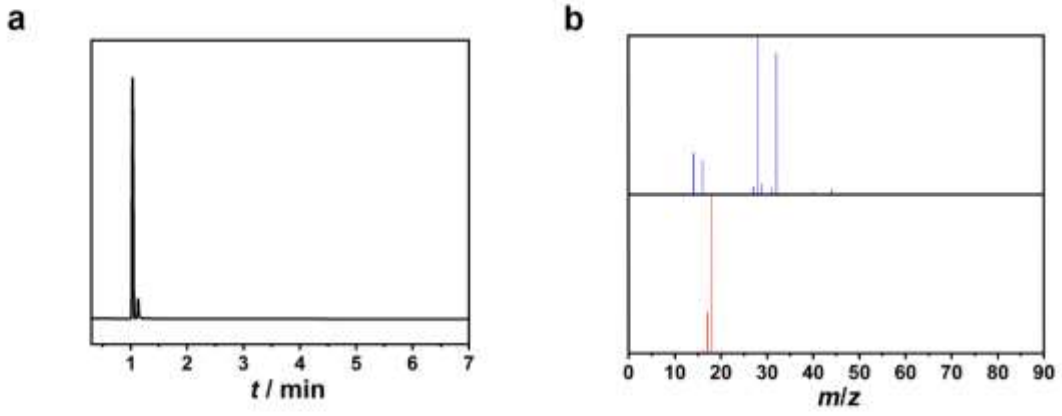

Fig. S16 | **a**, GC and **b**, MS spectra of gas released from half-electrolysis in alkaline salt water over 50 cycles. Top and bottom panels of MS corresponding to two peaks of GC in order.

Cyclic performance of half-electrolysis in Na_2SO_4 electrolyte.

When the electrolyte was changed to 1.0 mol L^{-1} Na_2SO_4, different HER and OER current densities were applied to investigate the cyclic performance of the half-electrolysis cell. The HER current density of 10.0 mA cm^{-2} was utilized to charge the AC electrode over 200 cycles. In order to accelerate the recovery of the half-electrolysis cell, the OER current density was changed from 10.0 to 39.1 mA cm^{-2} to discharge the AC electrode since the 101th cycle. **Fig. S16** and **S17** show that the HER branches became nearly overlayed since the 10th cycle, while all the OER branch repeated in each cycle. The ideal repetition of the OER branches at the larger current density indicates that the half-electrolysis cell can rapidly and reversibly recover to restart HER due to the advantage of the capacitive AC electrode.

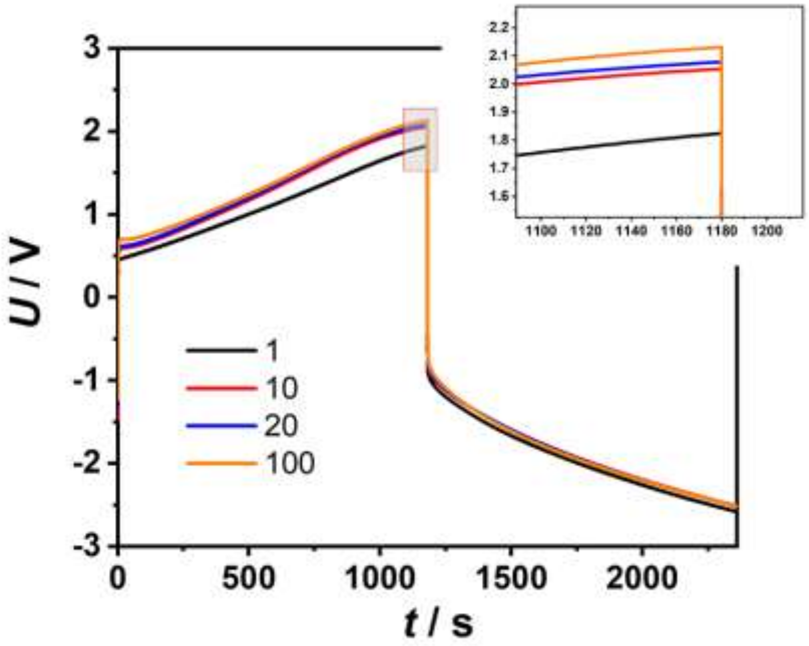

Fig. S17 | Galvanostatic electrolysis curves of half-electrolysis in 1.0 mol L^{-1} Na_2SO_4 electrolyte at 1st, 10th, 20th, 100th cycle at 10.0 mA cm^{-2}.

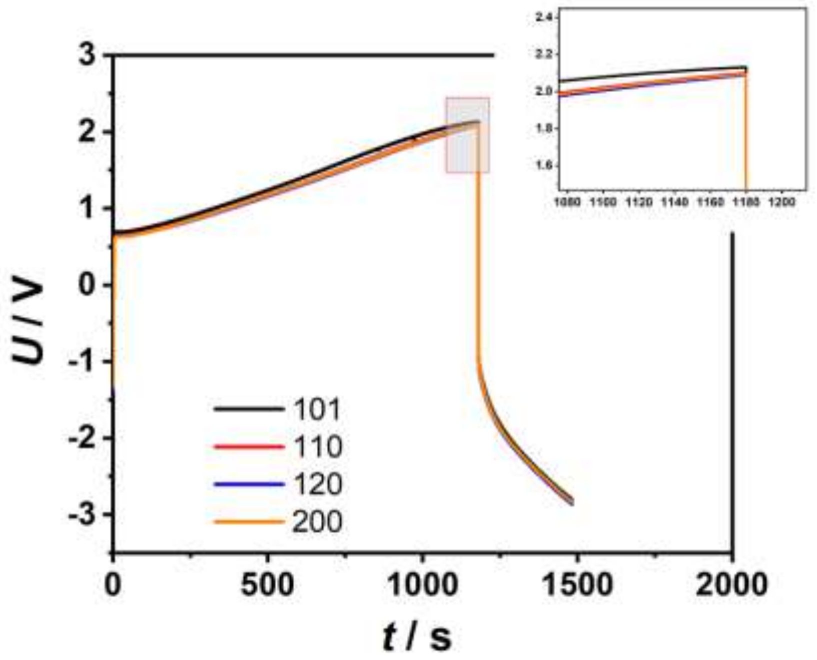

Fig. S18 | Galvanostatic electrolysis curves of half-electrolysis in 1.0 mol L^{-1} Na_2SO_4 electrolyte at 101st, 110th, 120th and 200th cycle at charging/HER current density of 10.0 and discharging/OER of 39.1 mA cm^{-2}.

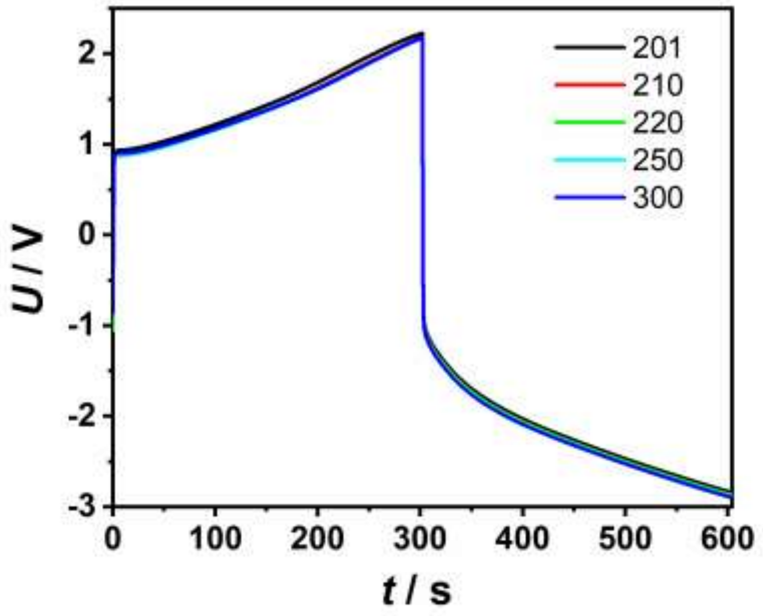

Fig. S19 | Galvanostatic electrolysis curves of half-electrolysis in 1.0 mol L^{-1} Na_2SO_4 electrolyte at 201st, 210th, 220th, 250th and 300th cycle at 39.1 mA cm^{-2}.

Cyclic performance of half-electrolysis in H_2SO_4 electrolyte.

In the case of the half-electrolysis of 1.0 mol L^{-1} H_2SO_4 electrolyte, an SCE electrode was applied to monitor and control the potential range of the AC electrode. The HER current density of 10 mA cm^{-2} and the potential range from −0.2 to 0.8 V *vs*. SCE were utilized to charge the AC electrode over 200 cycles. The curves of different cycles overlayed well, except for the initial ones before the 101th cycle (**Fig. S18** and **S19**). The similar reversible performance at the OER current density of either 10.0 or 40.0 mA cm^{-2} confirms the expected high electrochemical stability of the capacitive AC electrode in the half-electrolysis. As shown in **Fig. S20**, after 100 cycles, the capacitance retention was 96% of the initial capacitance and the coulombic efficiency gradually increased from the initial 90 to 100%. The slightly low initial coulombic efficiency may be attributed to the ion rearrangement, during the initial dis-/charging cycles, inside the pores of the AC electrode with a relatively high AC loading of 6 mg cm^{-2}. It is worth noting that since the OER current density utilized to discharge the AC electrode was changed from 10.0 to 40.0 mA cm^{-2}, the capacitance retention at the 200th cycle had turned to be 100% of the capacitance at the 101th cycle and the coulombic efficiency gradually increased from 92% to 98%.

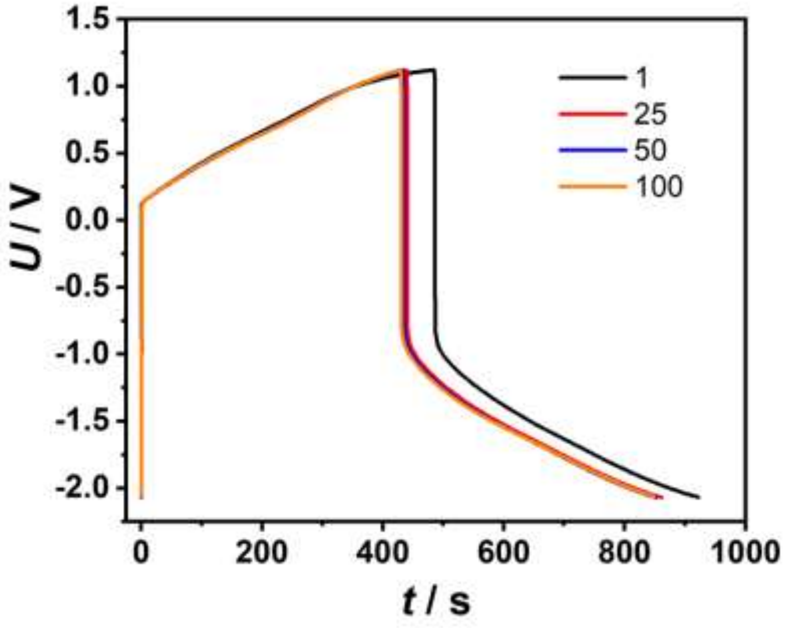

Fig. S20 | Galvanostatic electrolysis curves of half-electrolysis in 1.0 mol L^{-1} H_2SO_4 electrolyte at 1st, 25th, 50th, 100th cycle at 10.0 mA cm^{-2} with aid of SCE.

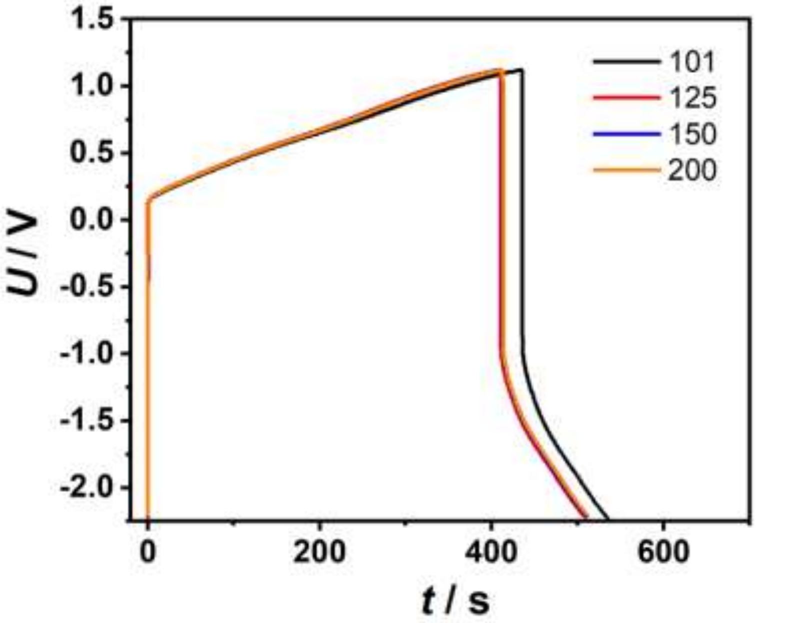

Fig. S21 | Galvanostatic electrolysis curves of half-electrolysis in H_2SO_4 electrolyte at 101st, 125th, 150th, 200th cycle at charging/HER current density of 10.0 and discharging/OER of 40.0 mA cm^{-2} with aid of SCE.

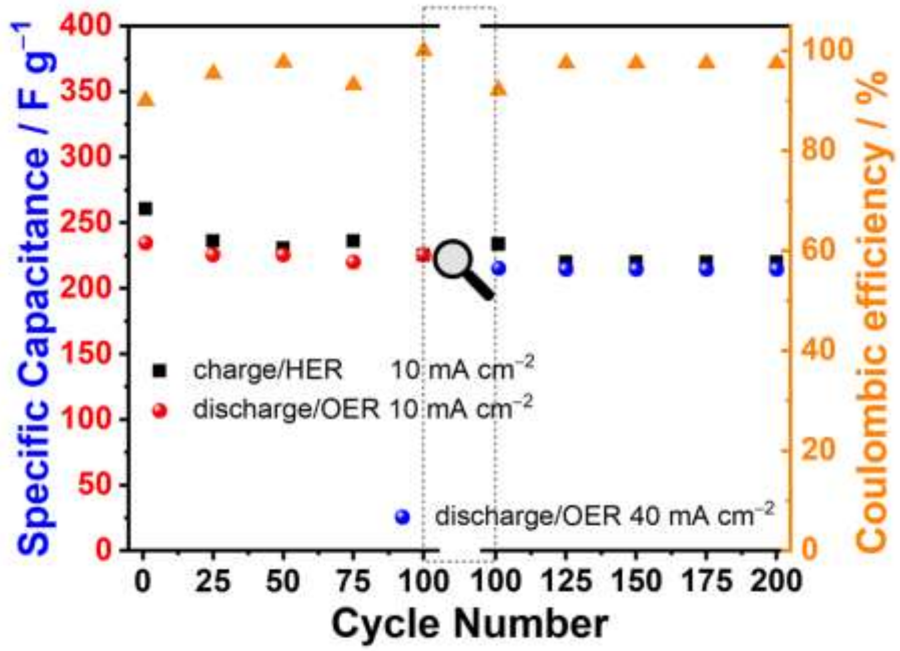

Fig. S22 | Specific capacitance and coulombic efficiency of AC electrode with cycles in half-electrolysis cell containing 1.0 mol L^{-1} H_2SO_4 electrolyte.

TEM image of Pt/C.

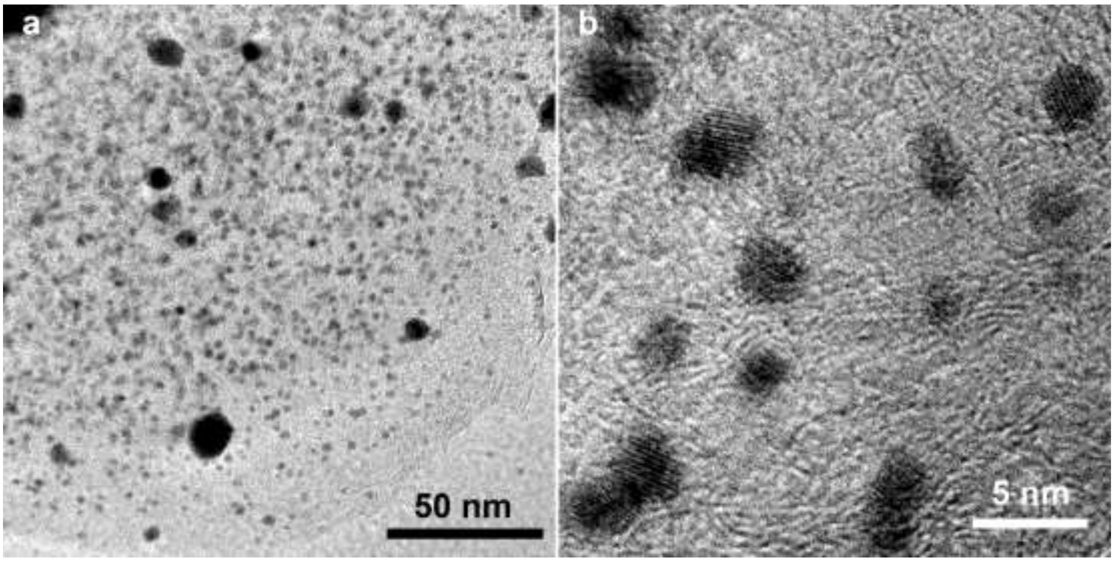

Fig. S23 | TEM images of Pt/C in **a**, low and **b**, high magnification.

Voltammetry curves and Tafel slopes of Pt/C and Pt wire.

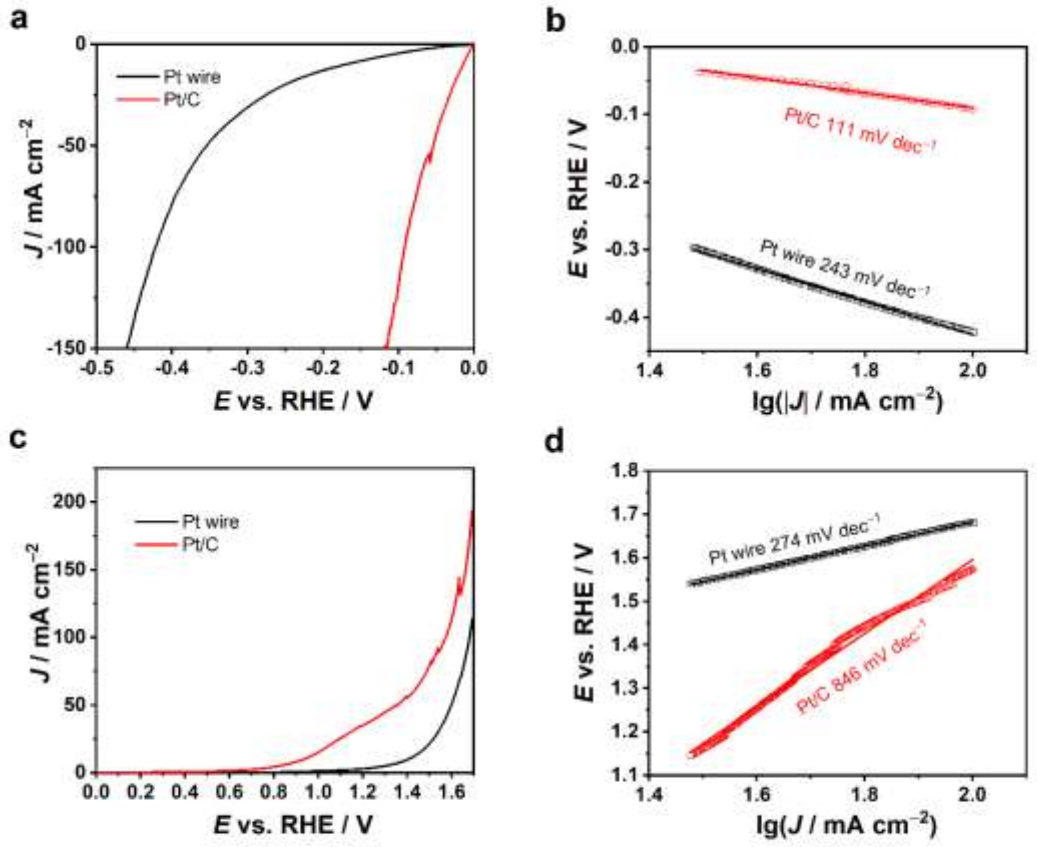

Fig. S24 | *IR*-corrected voltammetry curves for **a**, HER and **c**, OER, Tafel slopes for **b**, HER and **d**, OER of Pt/C and Pt wire.

EIS of half-electrolysis cell consisting of Pt/C and Pt wire.

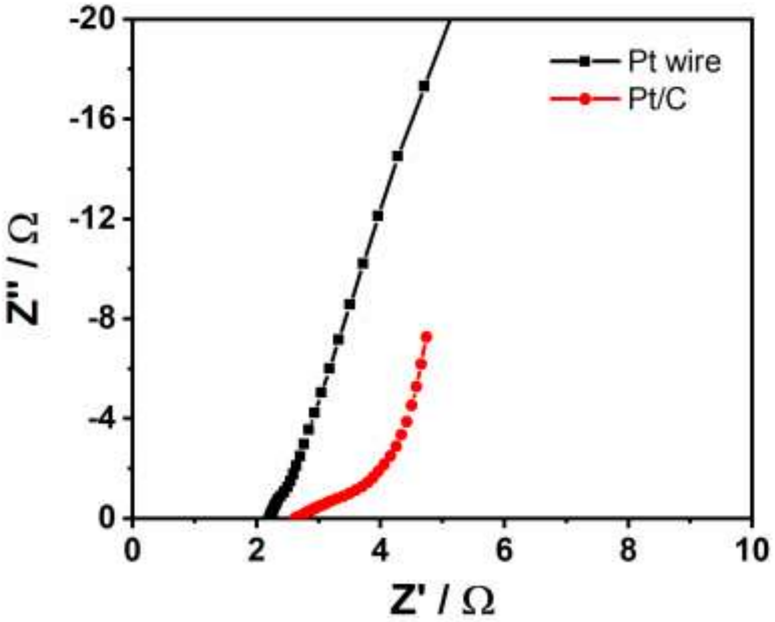

Fig. S25 | EIS spectra of half-electrolysis cell consisting of Pt wire and Pt/C.

Analysis of gas and reaction in half-electrolysis with Pt wire electrode in alkaline salt water.

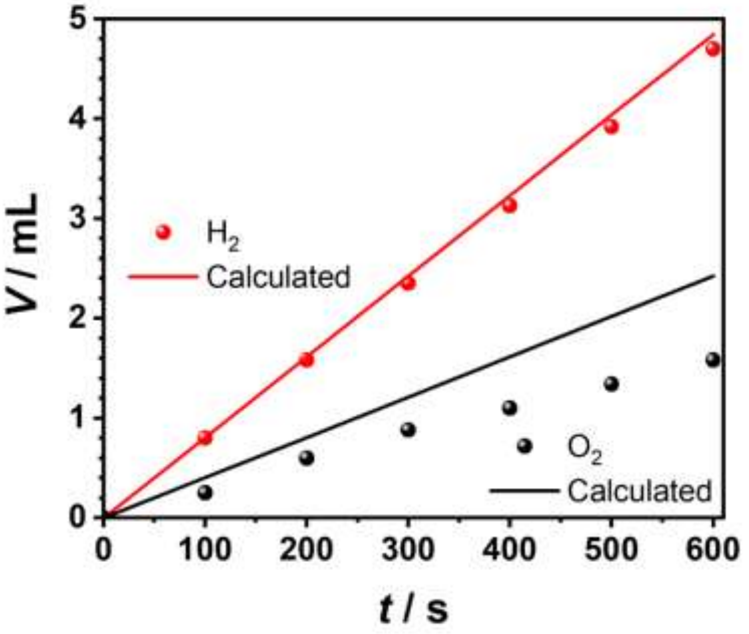

Fig. S26 | Volumes of H_2 (red dots) and O_2 (black dots) collected with a burette (markers) at different times, and calculated volumes (lines) from the charge passed during half-electrolysis in alkaline salt water on Pt wire electrode.

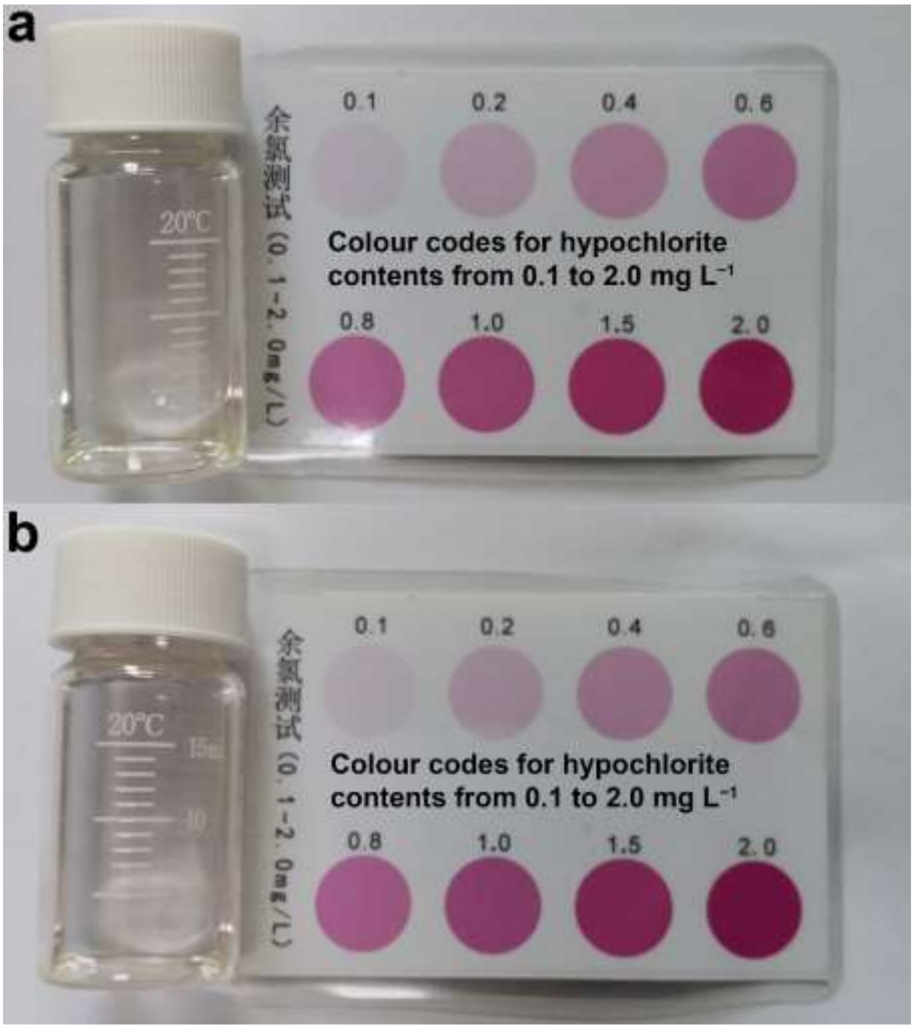

Fig. S27 | Digital photos of hypochlorite tests with DPD reagent for (0.5 mol L^{-1} NaCl + 0.5 mol L^{-1} KOH) electrolyte **a**, before and **b**, after OER in half-electrolysis on Pt wire electrode.

Analysis of reaction in half-electrolysis with Pt/C electrode in alkaline salt water.

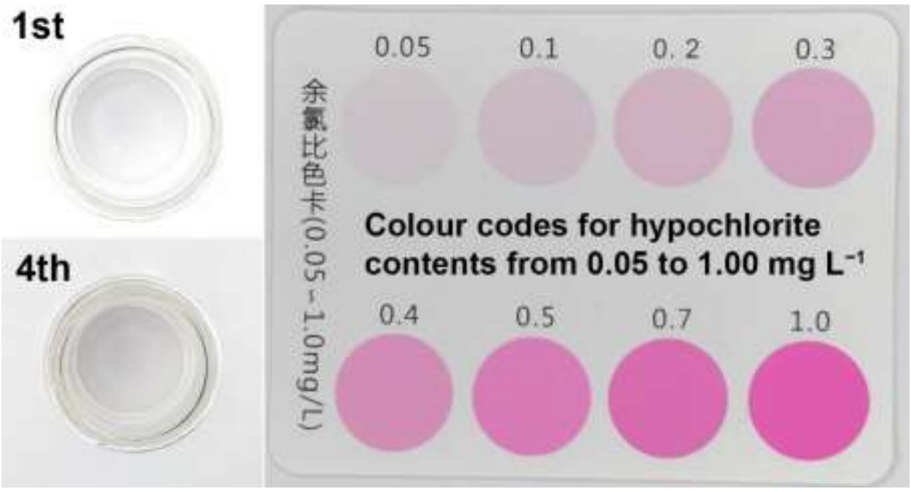

Fig. S28 | Digital photos (left, top view of the glass vial containing sample solution) of hypochlorite tests with DPD reagent for electrolyte of (0.5 mol L^{-1} NaCl + 0.5 mol L^{-1} KOH) after 1st and 4th OER in half-electrolysis on Pt/C electrode.

Comparison of half-electrolysis to decoupling of water splitting.

Table S1 | Comparison of half-electrolysis to decoupling of water splitting by faradaic reaction electrodes.

Buffer Electrodes	HER Electrodes	OER Electrodes	pH	J_{max} / mA cm^{-2}	Max Time	Efficiency / % HER	Efficiency / % OER	Energy Saving	Ref.
AC	Pt wire	Pt wire	0–14	195	300 cycles / 123 h	97.0	65.2	14%	This work
	Pt/C	Pt/C				98.0	96.3		
$Ni(OH)_2$	Pt	RuO_2/IrO_2	14	100	6.7 h	94.7	—	—	(14)
$Ni(OH)_2$	Ni	$Ni_{0.9}Co_{0.1}(OH)_2$	14	200	100 cycles / 2.7 h	100	—	—	(18)
polyaniline	Pt	RuO_2/IrO_2	0.3	50	40 cycles	100	100	—	(27)

Reference:

(S1) Fic, Krzysztof; Lota, Grzegorz; Meller, Mikolaj; Frackowiak, Elzbieta. Novel insight into neutral medium as electrolyte for high-voltage supercapacitors. *Energy Environ. Sci.* **2012,** *5* (2), 5842-5850.
